# Supplementary material for: Are carbon emissions trading and green financial instruments synergistic? -Comprehensive quantitative research based on content analysis
Source: PLoS One. 2024 Mar 7;19(3):e0298601. doi: 10.1371/journal.pone.0298601 (PMC10919601; doi:10.1371/journal.pone.0298601)
Supplement: S1 Data — (ZIP) [file pone.0298601.s001.zip › Data/地级市Z_F工作报告.zip!/╕╩╦α/╠∞╦«╩╨/└┤╫╘╣1⁄2╓┌║┼í╛┬φ┐╦╩2╛▌═°í┐_2019.docx]

各位代表：

　　现在，我代表市人民政府向大会作工作报告，请予审议，并请各位政协委员和其他列席人员提出意见。

　　一、2018年政府工作回顾

　　2018年，在省委、省政府和市委的坚强领导下，市政府坚持以习近平新时代中国特色社会主义思想为指导，认真学习贯彻党的十九大精神，全面落实习近平总书记视察甘肃重要讲话和“八个着力”重要指示精神，按照省第十三次党代会和市第七次党代会的总体部署，统筹推进稳增长、促改革、调结构、惠民生、防风险各项工作，较好地完成了市七届人大三次会议确定的目标任务。

　　经济运行平稳增长。认真贯彻中央和省上稳增长各项政策措施，全面落实“三重”“三一”工作机制，强化分析调度、强化要素保障、强化督促检查，全市经济运行稳中有进、稳中向好。预计实现生产总值640亿元，同比增长6.5%，其中，第一产业增加值86亿元，增长4.5%；第二产业增加值198亿元，增长5%；第三产业增加值356亿元，增长8%。规模以上工业增加值增长7%。固定资产投资增速降幅收窄，预计下降1.5%左右。社会消费品零售总额270亿元，增长9%。一般公共预算收入46.6亿元，同口径增长8%。城镇居民人均可支配收入26704元，增长8.5%；农村居民人均可支配收入7700元，增长9%。存贷款余额分别为1337.1亿元、957.83亿元，增长7.66%和14.43%。安全生产、节能减排和环境质量指标均在控制目标以内。

　　脱贫攻坚成效明显。坚持精准方略，聚焦深度贫困，紧盯“两不愁、三保障”目标，落实“五个一批”脱贫举措，预计全年减贫10.65万人，425个贫困村实现脱贫，甘谷、武山两县整县“摘帽”。完善落实“一户一策”，帮助11.67万户群众制定“一户一策”脱贫计划。突出抓好产业扶贫，制定出台“1+6+1+1”实施方案，推进农业保险“扩面增品提标降费”工程，落实到户产业扶持资金9.14亿元，贫困村新建果园11.35万亩，种植蔬菜7.2万亩、中药材7.17万亩，饲养畜禽24.8万头（只）；输转贫困劳动力5.1万人，创劳务收入9.96亿元；贫困村新增龙头企业68家，新建农民专业合作社2157个，建成“扶贫车间”156个、果蔬保鲜库87座。精准落实扶贫政策，实施建档立卡贫困户易地搬迁1672户8159人，完成危房改造8666户，建成行政村幼儿园368所，贫困人口慢性病签约管理5.96万人、重病兜底3271人。推进电商扶贫、构树扶贫、光伏扶贫，累计建立贫困村电商服务点1028个，种植构树8620亩，清水县30兆瓦和武山县23.7兆瓦光伏扶贫电站建成并网发电。探索创新扶贫模式，建立“千名县级干部联千村”联络员制度，设置村级“八大员”服务性岗位13292个，分两年筹措6.02亿元对全市农村安全饮水工程进行全面巩固提升，全省“扶贫车间”建设暨就业扶贫现场推进会、蔬菜产业扶贫现场推进会在我市召开。加大脱贫攻坚投入，争取中央和省上财政专项扶贫资金13.31亿元，天津市和5个中央定点帮扶单位分别落实帮扶资金1.87亿元、2130万元，中央定点单位协调落实项目资金3.4亿元；累计发放“双助贷”15.75亿元、特色产业发展贷款20.5亿元。

　　重大项目进展顺利。严格落实项目建设清单管理、领导包抓等制度，优化投资项目审批管理流程，持续推进联审联批工作，两次组织开展项目督查检查活动，重点项目建设有序推进。901个清单项目完成投资420.47亿元，62个重大前期项目扎实有效推进，75个市级领导包抓重大项目完成投资145.74亿元。天水火车站改扩建、天水汽车西站、甘谷客运中心全面建成，天水南站综合交通枢纽及站前广场正式启用，城区引洮供水主体工程全线贯通，天平高速公路天水段、天定高速藉口出口、秦州大道、麦积山大道、国道310线秦州至武山升级改造工程、曲溪城乡供水及麦积区潘集寨至街亭出口、甘泉至麦积山景区、武山县洛门至水帘洞景区3条旅游公路加快建设。甘肃（天水）国际陆港项目启动实施，落实省级专项补助资金5亿元，详规编制等工作进展顺利。岷山厂三阳川隧道开工建设。天水军民合用机场迁建、天水南站新增动车组存车线、静宁至天水高速公路天水段、天水绕城高速等项目前期工作取得实质性进展。

　　产业发展步伐加快。坚持不懈推进“一产调结构、二产扩总量、三产强基础”，产业发展呈现总量扩大、结构优化、效益提升的态势。重视发展十大绿色生态产业，建立“1+1+10”政策体系，实施绿色生态产业项目123个，完成投资98.05亿元。大力发展农业特色产业，果品总面积350万亩，产值87.5亿元；蔬菜种植面积135.1万亩，产值88亿元；新（改扩）建标准化规模养殖场80个，畜禽饲养总量2675万头（只），产值85.1亿元；输转农村劳动力69.28万人，创劳务收入145.6亿元；种植中药材40.23万亩，产值20.31亿元。粮食总产115.77万吨，增长2.19%。建成乡村振兴示范点108个。深入实施工业强市战略，六大传统产业加快转型，五大新兴产业开局良好。华天电子科技园、长城电工产业园一期全面建成，岐黄中医药产业园、装配式建筑产业园、电工产品综合检测实验研究基地、祁连山集团武山水泥技改迁建等项目进展顺利。冀城产业园规划编制完成，长城果汁、海林轴承、岷山机械搬迁改造有序推进，张家川县经济开发区升级为省级开发区，海林轴承、二一三电器、陇鼎建设工程、三和数码测绘入选全省战略性新兴产业骨干企业。加快发展文化旅游首位产业，制定出台《关于加快建设文化旅游强市的实施意见》及系列配套政策，麦积山游客服务中心、天水大剧院、华昌欢乐城、百花小镇加快建设，麦积山自驾游房车露营基地、南山云端休闲露营公园、中梁樱花小镇等项目积极推进，麦积山景区与华侨城赋能管理服务项目顺利实施。创建全国休闲农业与乡村旅游示范县2个、旅游专业村20个，清水县被评为中国体育旅游目的地，秦州区李官湾村入围中国美丽休闲乡村。全年接待游客4219万人次，旅游综合收入253.3亿元，分别增长35%和36%。加强商贸物流基础设施建设，天水信合物流园、天水腾邦物流园建成，天水粮食仓储物流生态产业园、麦积华夏物流园、武山金鑫物流园、天水城市蔬菜仓储分拣中心建设有序推进。市县区电商服务中心全面建成，麦积区、清水县、张家川县被列为国家电子商务进农村示范县，全市电商交易额42.8亿元，增长20%。

　　改革开放持续深化。以庆祝改革开放40周年为契机，进一步加大改革开放力度，政府系统完成重点改革事项135项。扎实开展“深化放管服改革突破年”活动，积极推进“四办”改革和“一网、一门、一次”改革，大力开展减证便民行动，取消调整和下放行政许可项目28项，取消市级证明事项66项，公布市级“最多跑一次”事项647项。市政府政务大厅搬迁新址，一体化在线政务服务平台建成运行，政务服务事项网上可办率达到98.2%。规范运行“多证合一”改革，全面推开“证照分离”改革，企业开办时间压缩至5个工作日以内，新增市场主体23983户、注册资金318.88亿元，分别增长27.64%和42.72%。深化农村土地制度和集体产权制度改革，累计发放农村土地承包经营权证书60.92万份。全面推开农村“三变”改革，5.41万户11.27万农民通过土地、技术、劳务、自有资金入股变为股东，村级集体经济“空壳村”全面消除。深化国资国企改革，基本确立国有资产监管政策体系，市属国有企业实现分类定位、统一监管，市县区盐业体制改革全面完成。促进金融改革创新，组建金额达8亿元的甘肃金控天水融资担保有限公司，设立天水市环保投资基金和中小企业创业创新投资基金，四零七医院挂牌新三板，天水花牛苹果集团、秦安雪原果品公司被郑商所指定为“苹果期货交割库”。推进质量强市战略，新增中国驰名商标1件、地理标志证明商标3件、甘肃省名牌产品15个。加强对外交流合作，制定完成《天水市关中平原城市群发展规划实施方案》，成功举办公祭伏羲大典暨第29届伏羲文化旅游节、2018绿色及可持续发展麦积山论坛、李杜诗歌节及龙舟、自行车、射箭、徒步等重大体育赛事，“李广杯”射箭等3项赛事被评为中国体育旅游精品赛事。组织华天科技等企业参加首届上海进博会，签订意向合同9.9亿元，外贸进出口总额达到42.5亿元，增长12%。组团赴广州、深圳、杭州、上海、西安、香港等地开展项目推介和招商引资，与广州天适集团、陕西白鹿仓集团、省公航旅集团等企业签订合作协议，全年共签约招商引资项目117项，实施99项，到位资金115.82亿元。

　　城乡建设扎实推进。坚持规划引领，《天水市城市总体规划纲要（2016—2035年）》编制完成，乡镇总体规划和行政村规划实现全覆盖。加强城市基础设施建设，新华路南延段、西大桥下穿道路、天润桥北立交、藉河南路西延段、成纪儿童公园、上亿广场、秦州印象•文景园步行街、秦州热源厂二期、天水固体垃圾处理循环产业园建成，有轨电车示范线一期、二十里铺大桥及接线工程、藉河生态治理二期、成纪新城地下净水工程、天水郡十字交通疏解、天水火车站广场地下人防及商业街、天水古城西关片区保护与利用等项目加快建设。实施优化城市环境提升城市形象工程，完成民主路、青年南北路、建设路、羲皇大道秦州段和麦积区天河南路、马跑泉中心大道等城区主次干道改造提升，实施城区关键交叉路口交通组织优化项目23个、交通信号灯智能化改造24处，整修改造小巷道30条，新建、改建城区公厕60座、旅游厕所68座，新建停车位3791个，市一中和省工业学院门前天桥建成投入使用。加强城市园林绿化，藉河一期续建绿化景观工程完工，城区新增绿地15万平方米。深入推进全国文明城市创建，广泛开展文明市民教育和文明单位、文明校园、文明社区等建设。下功夫治理城区乱搭乱建抢搭抢建、岷山厂区违法建设、出租车客运行业乱象丛生等问题。完成东湖、西湖、南湖长途客运站整合搬迁，市容市貌不断改观。加快推进智慧城市建设，城区公共场所无线免费上网全覆盖，天水云计算大数据中心、市级社会信用信息共享交换平台、云上天水APP、智慧旅游一期、智慧环保一期和政务信息资源整合等项目全面建成，我市被列为2018年国家电信普遍服务（4G网络建设）试点城市。着力改善农村基础条件，实施农村饮水安全巩固提升工程85处，新修梯田12万亩，新建堤防60.7公里，发展高效节水灌溉面积5.67万亩，治理水土流失面积250平方公里，完成生态造林21.88万亩，建成农村公路965公里、县乡道安全生命防护工程425公里，清水县被命名为“四好农村路”全国示范县。认真落实农村人居环境整治三年行动实施方案，加快全域无垃圾治理，建立落实河长制、湖长制，分类推进农村生活垃圾污水治理、卫生改厕、村容村貌提升，创建省市级美丽乡村示范村39个、整洁村305个，全市非正规垃圾堆放减少98%，陈年垃圾实现清零目标。推进大气、水、土壤污染防治“三大战役”，狠抓环保督察问题整改落实，20蒸吨以下燃煤锅炉全部淘汰改造，建成一级煤炭市场8处、二级煤炭网点268处，完成“三改一补”5万户，两区建成区黑臭水体及污染沟渠基本实现清零，全年空气质量优良天数304天。

　　群众福祉不断提升。省列10项、市列10件民生实事全面完成。优先发展教育事业，城区教育资源提升“2521”计划加快推进，新建、改扩建城区学校27所，增加义务教育学位2.5万个，“超大班额”问题基本解决，“大班额”问题得到缓解；天水市职教园区启动建设，与德国爱科特教育集团签订联合办学意向书，市三中新校区、玉泉中学、玉城小学基本建成，农村中小学教师周转宿舍、农村边远地区中小学温暖工程顺利实施，改薄“20条底线”全部达标；全市文理二本以上上线10327人，上线率36.21%，较上年提高2.57个百分点。加快科技进步与创新，争取省列科技项目56项，组织实施各类科技项目436项，取得科技成果106项，天水经济技术开发区产业孵化园被认定为省级科技孵化器。实施文化体育惠民工程，市体育中心项目主体完工，新建文化馆、图书馆乡镇街道分馆246个、文化遗产“历史再现”工程博物馆11个、乡镇及社区体育健身中心8个、行政村农民体育健身工程210个。完成陇南书院、后街清真寺等保护修缮工程，创排完成舞台剧《天一生水》，《一画开天》实现旅游旺季常态化演出，影片《天水来的姑娘》在央视播出。继秦州电视台之后，麦积、清水、秦安、武山电视台实现与市电视台并网。深入推进公立医院综合改革，三级医疗机构全面推行药品采购“两票制”，参保居民在定点医疗机构就诊实现“一站式”即时结报，市中心血站整体搬迁等项目进展顺利。强化就业指导服务，全市城镇新增就业5.47万人，城镇登记失业率3.2%。加强保障性住房建设，实施棚户区改造25780户，为10365户低收入家庭发放住房租赁补贴1265万元。落实全民参保登记计划，机关事业单位养老保险改革全面完成，城乡居民社会养老保险参保率97.8%，待遇发放率100%，城市低保月保障标准提高7.6%，农村低保年指导标准提高6.3%。强化食品药品安全监管，推进安全生产隐患排查和重点领域专项治理，安全生产事故起数、死亡人数、受伤人数、直接经济损失分别下降16.5%、20.3%、30.2%、66.6%。加强耕地和矿产资源保护，“大棚房”问题专项清理整治行动扎实有效。全力抓好防灾救灾工作，受灾群众生活妥善安置，灾后恢复建设有序开展。深入推进“平安天水”建设，扎实开展扫黑除恶专项斗争，加大矛盾纠纷排查调处和社会治安综合治理力度，一批信访突出问题得到有效解决，社会大局保持和谐稳定，刑事案件和治安案件分别下降15.7%和24.3%，首次实现了当年命案全侦破。统计调查、档案史志、防震减灾、人民防空、双拥共建、民兵预备役、社会团体、妇女儿童、老龄老干、老年教育、残疾人事业和关心下一代等工作都取得了新成绩。

　　自身建设全面加强。全面落实省委、省政府和市委各项决策部署，自觉接受人大及其常委会法律监督和工作监督、政协民主监督、社会舆论监督，认真执行人大决议，充分吸纳政协意见，办理答复人大代表意见建议207件、政协提案237件。坚持依法行政，全面落实普法责任制，完成“七五”普法中期考核。积极推进地方立法工作，《甘肃省麦积山风景名胜区条例》《天水市饮用水水源保护区环境保护管理办法》公布施行，《天水市古树名木保护条例（草案）》已经市人大常委会审议通过。修订完善市政府工作规则，严格执行重大行政决策程序规定，召开市政府党组会议11次、理论中心组学习会议12次、常务会议20次、市长办公会议38次、市政府专题会议25次，行政决策的科学化、民主化、法治化水平不断提升。重视加强电子政务建设，市县区两级政府和市政府部门无纸化办公实现全覆盖，会议文件进一步压减，政务效能明显提升。严格落实中央“八项规定”及实施细则精神和省市《实施办法》，深入开展“转变作风改善发展环境建设年”“政务诚信建设年”活动，扎实推进“两学一做”学习教育常态化制度化，认真落实政府党风廉政建设主体责任和领导干部“一岗双责”，严格执行省政府“十不准”规定，政风行风不断好转，政府执行力和公信力持续增强。

　　各位代表，刚刚过去的一年，面对复杂多变的外部经济环境和低温暴洪等自然灾害造成的不利影响，全市上下积极主动作为，聚力攻坚克难，在决胜全面建成小康社会、建设幸福美好新天水的征程上迈出了坚实一步。能够取得这样的成绩，是省委、省政府正确领导、大力支持的结果，是市委统揽全局、坚强领导的结果，是市人大及其常委会、市政协和社会各界全力支持、有效监督的结果，是全市干部群众齐心协力、团结奋斗的结果。在此，我代表市人民政府，向各位人大代表、各位政协委员，向奋战在全市各条战线上的广大干部群众，向各民主党派、人民团体、工商联和无党派人士，向离退休老同志，向天水籍在外人士，向驻市人民解放军、武警官兵和中央、省属驻市单位，向关心支持天水发展的社会各界人士，表示崇高的敬意和衷心的感谢！

在充分肯定成绩的同时，我们也清醒地认识到，市政府工作还存在许多问题和不足。主要是：虽然大部分经济指标增速高于全省平均水平，但固定资产投资、城乡居民人均可支配收入没有达到市七届人大三次会议确定的预期目标；产业发展水平有待进一步提升，农业产业组织化、品牌化程度不高，工业经济结构不优、新兴工业项目较少，文化旅游资源优势还没有真正转化成发展优势；项目建设前期审批手续多、周期长，新开工的大项目、好项目不多，一些在建项目没有达到进度要求，招商引资项目落地见效较慢；城市基础设施和公共服务领域还有许多欠账和短板，群众关心关注的一些热点难点问题还没有得到有效解决；深化“接放管服”改革还不到位，推进“四办”改革、实现“一网通办”和“最多跑一次”存在一些“堵点”“难点”，企业和群众办事还存在不方便的地方；一些政府工作人员能力素质不能适应新时代要求，服务发展的意识不强，促进经济社会发展的办法不多、思路不广，不作为、慢作为问题仍然存在，不落实、落不实现象时有发生，政府工作的公信力和执行力仍需强化，改进工作作风、营造良好环境还需持续发力。对于这些问题和不足，我们将高度重视，努力加以克服解决。

　　二、2019年主要目标和重点工作

　　2019年，是中华人民共和国成立70周年，也是打赢脱贫攻坚战、决胜全面建成小康社会的关键之年。今年政府工作的总体思路是：以习近平新时代中国特色社会主义思想为指导，深入贯彻党的十九大精神，全面落实习近平总书记视察甘肃重要讲话和“八个着力”重要指示精神，按照省委、省政府和市委的部署要求，坚持稳中求进、争先进位的工作总基调，以五大新发展理念为引领，以供给侧结构性改革为主线，以推动高质量发展为目标，以十大绿色生态产业为抓手，继续打好三大攻坚战，千方百计稳增长，统筹兼顾促改革，坚定不移调结构，全力以赴惠民生，多措并举防风险，推动全市经济社会持续健康发展，为与全省全国一道全面建成小康社会打下坚实的基础。

　　全市经济社会发展的主要预期目标是：生产总值增长6%，规模以上工业增加值增长6.5%，固定资产投资增长6%，社会消费品零售总额增长8%，一般公共预算收入增长7%，城镇居民人均可支配收入增长8.5%，农村居民人均可支配收入增长9%，居民消费价格指数涨幅控制在2.5%以内，城镇登记失业率控制在4%以内，环境质量和节能减排指标均控制在省上下达的目标以内。

　　围绕上述目标，市政府将重点抓好以下八个方面的工作：

　　（一）持续深入打好三大攻坚战

　　打好三大攻坚战，是全面建成小康社会必须跨越的关口，既要紧盯全面小康目标持续用力，又要瞄准阶段任务集中攻坚，以更大力度推动三大攻坚战取得新成效。

　　以“绣花功夫”持续推进脱贫攻坚。围绕“两不愁、三保障”目标，夯实政策落实、产业脱贫、精准帮扶、各方责任、基层队伍和工作作风基础，坚持质量标准，用心用情用力做好脱贫攻坚工作，确保全年减贫11.6万人，490个贫困村退出，秦州区、麦积区、秦安县、清水县、张家川县整县区“摘帽”。加快实施产业扶贫方案，精准对接“一户一策”，发挥到户产业扶贫资金效益，抓好果菜药薯牛羊等特色优势产业培育。高度重视龙头企业建设和专业合作社作用，强化产业组织化程度和品牌化效应，新增农业产业化龙头企业20家、农民专业合作示范社200家、示范家庭农场100家，推动小农户与大市场有效对接。全面推广农业灾害保险和目标价格保险，扶持发展乡村旅游、电商扶贫、光伏扶贫，促进贫困群众稳定增收。加快实施自然村道路、危房改造、易地扶贫搬迁项目，年内全部消除农村危房，全面完成“十三五”易地扶贫搬迁任务，全面完成农村饮水安全巩固提升任务，农村自来水普及率达到98%。积极推进健康扶贫、教育扶贫，认真落实贫困人口“先诊疗后付费”“一站式”结报和贫困家庭学生资助政策，年内建成贫困村幼儿园32所、标准化村卫生室40个，九年义务教育巩固率达到97.3%，贫困人口家庭医生签约服务实现全覆盖。加快实施就业扶贫三年行动计划，落实职业培训补贴政策，开展精准扶贫劳动力培训4.2万人，输转建档立卡贫困劳动力2.5万人。积极推广“扶贫车间”模式，继续做好村级服务性岗位开发，让更多群众通过就业实现稳定脱贫。注重扶贫与扶志、扶智相结合，引导贫困群众树立主体意识，着力解决农村“八差”问题，激发脱贫攻坚内生动力。加强东西部扶贫协作和中央单位定点帮扶，力争在产业开发、劳务协作、技术创新、人才交流等方面争取更多帮扶项目和资金。

　　以“铁腕手段”切实加强污染防治。全面贯彻习近平生态文明思想，严格落实天水市污染防治攻坚战实施方案，努力使天水的天蓝、地绿、水清、环境美好。坚决打赢蓝天保卫战，聚焦工业、燃煤、扬尘、机动车四大领域，突出抓好燃煤锅炉整治淘汰、城市集中供热改造、施工扬尘污染治理、柴油货车污染治理、工业窑炉治理等工作，统筹实施煤改气、煤改电或清洁煤替代工程，积极争取北方地区清洁取暖城市试点，全市空气质量优良天数比例达到83%以上。着力打好碧水保卫战，认真落实河长制、湖长制，以渭河、葫芦河、牛头河等河流为重点，扎实开展河湖清“四乱”专项行动和城市黑臭水体专项整治，分门别类推进城乡污水收集处理，完成县区污水处理设施提标改造，开展集中式饮用水水源地环境保护专项行动，境内河流考核断面水质达标率、县级以上集中式饮用水水源地水质考核达标率均达到100%。扎实推进净土保卫战，完成农用地土壤污染状况详查，强化土壤污染管控与修复，废旧农膜回收利用率稳定在80%以上，畜禽养殖废弃物综合利用率达到73%以上，实现农药化肥使用零增长，保障全市土壤环境安全。坚持对环境违法行为“零容忍”，扎实推进环保督察问题整改落实，支持推动环境保护公益诉讼，坚决遏制环境违法行为。坚持不懈加强生态建设，继续推进新一轮退耕还林、天然林保护等重点生态工程，加快大规模国土绿化，完成生态造林20万亩以上。

　　以“底线思维”防范化解重大风险。认真落实中央和省上防范化解地方政府债务和隐性债务的部署要求，进一步规范政府举债行为，坚决遏制隐性债务增量，多渠道化解隐性债务存量，提高政府性债务规范化管理水平。坚持开前门、堵后门，充分发挥政策性担保公司和融资平台公司作用，通过争取新增债券、设立政府投资基金、出让国有资源资产、资产证券化、规范实施PPP项目等多种渠道，保障重大项目建设资金需求。强化金融领域监管，做好各类扶贫贷款的回收续贷，严厉打击民间社会非法金融活动，加大金融风险宣传教育，推进社会信用体系建设，牢牢守住不发生区域性金融风险的底线。

　　（二）坚定不移实施项目带动战略

　　牢固树立发展是第一要务、抓项目就是抓发展的理念，把项目建设作为解决一切发展问题的金钥匙，扎实做好项目的论证储备、跟踪争取和组织实施工作，持续扩大有效投资规模。

　　着眼夯实基础抓项目。围绕打造交通枢纽、强化供水保障、重视市政建设，着力抓好打基础、利长远的重大项目。年内建成天定高速藉口出口、秦州大道、麦积山大道、国道310升级改造和3条重点旅游公路，城区引洮供水工程建成通水，加快实施天平高速公路天水段、引洮供水二期天水配套、曲溪城乡供水等项目，开工建设天水机场迁建试验段、天水南站新增动车组存车线、静宁至天水高速公路天水段、甘肃（天水）国际陆港基础设施、三阳川罗家沟隧道及引线工程，启动天水南站片区二期工程，积极推进天水绕城高速、310国道麦积至秦州段改线提升工程、兰州至天水至汉中城际铁路、陇海铁路与宝兰客专连接线、天水至陇南铁路等项目前期工作。

　　立足产业发展抓项目。按照改造提升传统产业、培育壮大新兴产业的要求，紧盯十大绿色生态产业发展，重点抓好列入全省绿色生态产业发展规划的59个重点项目建设，加快推进电工产品综合检测实验研究基地、岐黄中医药产业园、三峡新能源清水白驼60兆瓦风电、清水50兆瓦村级光伏扶贫电站、太极阿胶三期、甘肃伏羲药业、麦积山游客中心、新天德绿色新材料产业园、甘肃移动第二大数据中心等重大示范性工程，积极谋划冀城现代高载能循环经济产业园、中子技术产业园、数字经济产业园，促进文化旅游、航空维修制造、装配式建筑、生态康养、数字信息等产业取得新进展，以绿色生态产业发展助推高质量发展。

　　紧盯招商引资抓项目。充分发挥比较优势，利用各类园区平台，加强与央企和国内500强企业的对接洽谈，积极开展精准招商，力争引进一批新的延链、补链、强链项目。牢固树立“你投资、我服务，你发财、我发展”的理念，认真落实招商引资优惠政策，构建“亲”“清”新型政商关系，狠抓各类签约项目和意向项目跟踪服务，全力促成天水白鹿仓国际旅游度假区、天适集团樱花小镇、万宝集团无人机、绿地集团康养小镇、恒大集团旅游小镇和江南酒店、麦积山大酒店等五星级酒店项目尽快落地实施，确保项目招得来、落得下、实施好。

　　强化服务保障抓项目。全面推行代办制、联审联批、并联审批，优化项目审批流程，建立十大生态产业审批绿色通道，最大限度缩短审批时限，破解项目建设“四难一低一慢”问题，实现投资项目全流程审批时间压缩一半、企业投资项目核准时限压缩到10个工作日以内。继续实行重大项目领导包抓、督查检查、稽察通报等制度，重视项目入库统计，开展项目招投标领域专项整治，严把工程施工、安全监管、竣工验收、投资审计等关口，确保项目建设质量过硬、推进有力、安全快速。

　　（三）始终坚持农业农村优先发展

　　坚持把乡村振兴战略作为“三农”工作的总抓手，全面落实乡村振兴战略规划，全力以赴抓重点、补短板、强弱项，加快推进农业农村现代化。

　　大力发展现代农业。落实藏粮于地、藏粮于技战略和粮食安全责任制，全年粮食总产稳定在110万吨以上。紧盯打造果品、蔬菜、畜牧、劳务四个百亿元主导产业目标，扎实推进果业品牌增效、蔬菜技改增效、畜牧扩链增效、劳务技能增效计划，加大冷链物流、仓储设施、终端市场、产业保险建设，积极构建现代农业产业体系，年内新建果品基地10万亩，蔬菜种植面积稳定在135万亩，新建和提升改造标准化规模养殖场100个，新建果蔬保鲜库68座，劳务输转69万人次，果品、蔬菜、畜牧产值和劳务收入分别达到110亿元、95亿元、95亿元、159亿元以上。以推进农业供给侧结构性改革为主线，提升2个省级现代农业示范区和10个省市级现代农业示范园建设水平，高标准、高质量抓好100个乡村振兴示范点建设。大力培育新型职业农民，培养用好农村“土专家”“田秀才”“农创客”，年内开展农业科技培训15万人次以上，新认定新型职业农民3000人。

　　改善农村基础设施。深入推进“四好农村路”建设，全面推行农村公路“路长制”，实施县乡道路网改善工程152公里、安全生命防护工程941公里、“畅返不畅”整治项目959公里，重视抓好村组道路和产业路建设。加强水利基础设施建设，深刻汲取暴洪灾害教训，高度重视河道疏浚和治理工作。发展高效节水灌溉面积5.7万亩，治理水土流失250平方公里，新修梯田10万亩，新建堤防60公里。完善农村信息基础设施，加快“宽带乡村”和电信普遍服务4G网络建设，扩大农村地区光纤宽带覆盖范围。下功夫提升城乡电网质量，完成2108个行政村电网改造项目，促进配电网从“温饱型”向“小康型”升级。

　　抓好美丽乡村建设。学习借鉴浙江“千万工程”经验，深入开展农村人居环境整治三年行动，持续推进全域无垃圾治理，建立健全农村垃圾收集处理和污水处理机制，加强公共空间和庭院环境整治，加快推进农村“厕所革命”，切实解决垃圾围村围河现象，年内新建农村生活垃圾中转站3处、收集点192个，建成“美丽乡村”省市级示范村40个、整洁村305个。狠抓乡风文明治理，加大力度治理农村高价彩礼问题。充分发挥村级“八大员”作用，推进乡村公益设施村民共建共管共享。

　　（四）着力加快工业转型升级步伐

　　坚定不移实施“工业强市”战略，改造提升传统产业，大力培育新兴产业，加快产业集聚发展，促进工业提质增效、转型升级。

　　加快扩量提质步伐。充分运用数字信息和现代化手段改造提升传统企业，使其重振雄风，融入绿色生态产业。坚持“一企一策”，加大“10强50户”企业扶持力度，推动龙头骨干企业做大做强，新增规模以上工业企业10户以上。实施投资千万元工业项目100个以上，加快华天集成电路高密度封装产业升级、天光节能系列器件产品研发与产业化、长开中压开关设备个性化定制系统、长城果汁年产10万吨果蔬汁生产线、六九一三通讯设备生产和维修保障服务基地、孟姑发酵食品及手工挂面苦荞加工、福美软瓷新材料、甘谷脊兽建材产业园、霞宇科技智能化节水等项目建设。围绕发展五大新兴产业，抓好装配式建筑产业园、大型客机改货机、武山县无干扰地岩热源供热等项目，支持中航天飞争取维修基地项目落地建设，打造省级军民融合创新示范区，培育工业发展新动能。

　　重视发展园区经济。加快天水经济技术开发区扩区增容步伐，以托管方式理顺管理体制，加快冀城产业园建设，增强园区产业聚集能力，吸引更多项目入驻园区，开发区工业增加值增长7%以上。全面落实支持城区工业企业搬迁改造政策措施，加快推动长城果汁、海林轴承、岷山机械等企业搬迁改造，促进工业化和城市化协调发展。支持县区工业园区建设，加强园区基础配套和要素保障，推动秦州区经济开发区、武山县工业园区升级为省级开发区。

推进企业技术创新。实施创新驱动战略，支持重点企业加快装备升级改造，创建省级以上企业技术中心和工程中心2家、省级以上技术创新示范企业2家。充分利用国家重点实验室、国家级质量监督检验中心和省级以上企业技术中心等技术资源，提高重点工业企业核心竞争力，促进电子封装、高档数控机床、电工电器、智能仪表等新产品达到国内一流、国际领先水平。加强产业工人队伍建设，积极培育工匠和技能人才，鼓励支持职工创新创造。深入推进质量强市示范城市创建，加大商标品牌培育保护，年内新增注册商标600件以上，新增中国驰名商标1件。

　　（五）做大做强文化旅游首位产业

　　旅游业不仅是现代社会蓬勃发展的朝阳产业，而且也是融合一、二、三产发展的可重复消费的绿色产业。我市厚重的文化历史底蕴、良好的生态气候环境、优越的区位条件、众多的人口资源对于发展文化旅游业具有得天独厚的优势。要全面落实《关于加快建设文化旅游强市的实施意见》，加快文化旅游软硬件建设，推动文化、旅游、康体、养生、养老等产业融合发展，全面提升文化旅游业发展质量和效益。

　　推动文化旅游融合。按照建设大景区、开拓大市场、发展大旅游、做强大产业的思路，继续推进伏羲文化旅游产业园、麦积山文化旅游产业园、卦台山文化旅游产业园、大地湾文化旅游产业园建设，启动建设白鹿仓国际旅游度假区，加快华昌欢乐城、麦积山自驾游房车露营基地、黄河雕漆文化博览园、李官湾乡村旅居体验园、天水大剧院、伏羲庙游客中心及停车场、南郭寺景区游客中心、东柯杜甫文化主题园、工业博物馆等重点文化旅游项目建设。扶持飞天雕漆、黄河雕漆、汉唐陶艺、大漠行麻编鞋业、新天丝毯等文化企业做大做强，举办文化旅游商品创意设计大赛和展销大会，加快文化旅游商品集散中心建设，推动文化旅游产业持续快速发展，旅游人次和综合收入均增长20%以上。

　　大力发展乡村旅游。认真落实《关于加快乡村旅游发展的实施意见》，编制乡村旅游发展规划，抓好国家和省列15个乡村旅游示范村、10个旅游扶贫重点村建设，为全市乡村旅游发展树立典型、打造样板。突出“土气、老气、生气、朝气”，挖掘利用特色文化资源，深入实施“旅游名镇、名村、名景、名店”打造工程，规划建设大象天水西秦古镇和南河川、党川花庙河等田园旅游综合体，扶持发展“一村一品”和“一家一艺”特色乡村旅游产品，年内建成乡村旅游专业村7个、创建星级农家乐15户，乡村旅游接待人数和综合收入分别增长25%和30%以上。

　　促进消费提质升级。加快商贸流通基础设施建设，做好天水商贸城、会展中心招商经营工作，加快秦州藉口商贸城、武山洛门森源蔬菜果品大型商品交易市场、秦安小商品城等项目建设，建成农产品产地批发市场1个，实施冷链物流项目4个。高度重视市县区便民市场建设，新建、改造县乡农贸市场13个。举办“人文天水、养生美食”评选活动，推进餐饮街区、夜市建设和早餐惠民工程，培育一批连锁化品牌化餐饮企业，形成能满足多层次多样化消费需求的餐饮服务体系。大力发展电子商务，加快实施国家电子商务进农村综合示范项目，积极推广“烟铺模式”“清水模式”“秦安模式”，培育树立天水电商新模式和网货品牌，电子商务交易额增长20%以上。

　　（六）不断加快区域中心城市建设

　　围绕宜居宜业宜游城市定位，不断完善城市功能配套，提升城市发展内涵，加快新型城镇化进程，全方位提升建设管理水平。

　　完善城市综合功能。发挥天水市第四版城市总体规划引领作用，做好重大建设项目和民生项目规划服务。统筹推进“八大新城”建设，不断完善基础设施，提升承载能力。建成有轨电车示范线一期、藉河二十里铺大桥、天水郡十字交通疏解工程，加快藉河生态治理二期、岷山厂三阳川隧道、天水古城西关片区保护与利用等项目建设，启动实施藉河生态治理三期、天河广场地下空间综合利用、红桥南接线、藉河南沟河口大桥、罗峪河五中桥、渭河3号大桥、岷山生态文化公园，积极推进南北两山景观大道、秦麦高速公路市政化改造、成纪新城集中供热、麦积桥南集中供热扩建、颖川东柯新城集中供热、秦州污水处理厂综合开发、马跑泉主镇区改造、天水郡片区综合改造等项目。持续抓好城区示范路、整洁路、规范路建设，完成成纪大道西段、羲皇大道七里墩至潘集寨、皇城路、藉河北路提升改造，完善中心城区综合服务功能。

　　推进城市管理升级。深入开展全国文明城市、国家园林城市、国家卫生城市创建，加大对市民的文明宣传教育，广泛开展创建文明单位、文明校园、文明小区、文明街道等活动，全面落实“门前三包”责任制，新增公共绿地18万平方米，实现城区全天候保洁。全面推进优化城市环境提升城市形象工程，完成30个主要节点路口交通组织优化和12处交通信号灯智能化改造，严控严管各类违章建设，继续做好城区老旧小区改造、背街小巷综合整治、人行通道畅通、公厕建设改造、绿化美化亮化，加强流浪狗管理、城市“牛皮癣”整治、噪音污染治理，有效解决交通堵、停车难、秩序乱、环境差等“城市病”。加强出租车行业管理，规范网约车市场发展，提升保障能力和服务水平。加快智慧城市建设，扩大城区免费无线网络覆盖范围，启动实施智慧医疗、智慧教育、智慧交通等项目，建成智慧社区20个，提升城市管理智能化水平。加大宣传教育力度，引导市民和司机文明礼让，形成“车让人、人让车”的文明出行之风。

　　加快特色城镇建设。坚持把特色小镇建设作为推进新型城镇化的重要抓手，优化空间布局，完善基础设施，创新发展体制机制，聚焦特色产业、新兴产业、发展要素，构建“生态+产业+旅游+文化”融合发展格局，重点抓好秦州区樱花小镇、孙集文化小镇，麦积区街亭古镇、甘泉特色小镇，甘谷县礼辛风情小镇，秦安县刘坪桃园小镇，武山县山丹特色小镇，清水县秦亭小镇，张家川县马鹿特色旅游小镇建设。同时，高度重视解决部分乡镇脏、乱、差等问题。

　　（七）持续激发经济发展动力活力

　　坚持把改革开放作为推动经济发展的动力引擎，把大力发展县域经济作为补短板的重点，全面落实各项重点改革任务，全方位扩大对外开放，着力增强经济发展的内生动力、外向活力。

　　深化接放管服改革。持续加大简政放权力度，推进商事制度改革，着力“减证照、压许可”，深化“证照联办”“多证合一”改革，推行注册登记全程电子化，激发市场主体活力，全年新增市场主体9000户以上。深入推进“四办”改革，大力推行“互联网+政务服务”，优化提升政务大厅“一站式”功能，完善“前台综合受理、后台分类审批、统一窗口出件”政务服务模式，政务服务事项进驻政务大厅“应进必进”，实现70%以上事项“一窗”分类受理，90%以上事项网上可办。扎实开展营商环境突出问题专项整治，持续开展减证便民行动，简化办事流程，明确办结时限，做到“一次告知”，推动企业和群众办事线下“只进一扇门”，现场办理“最多跑一次”。

　　推进重点领域改革。全面完成政府机构改革，加快推进事业单位改革。分类推进国有企业改革，完成市属国有企业公司制改革，完善企业薪酬分配制度，推进混合所有制改革。理顺国有资产管理体制，实行经营性国有资产集中统一监管，初步形成“国资委—国投公司—国有企业”三级国有资产管理格局，促进国有资产保值增值。深化农村集体产权制度改革，完善推广农村“三变”改革，巩固集体林权制度改革成果。深化财税改革，严格落实预算管理改革政策，全面实施预算绩效管理，加快社会保险费和非税收入征管职责划转，进一步完善财政事权和支出责任划分。完善地方金融监管体制，创新运用多层次资本市场，积极推进城投债发行，做好企业首发上市、新三板和区域性股权市场挂牌工作。深化县域综合改革，认真研究县域经济发展的突出问题和困难，制定出台加快县域经济发展的实施意见，完善县域经济发展规划、特色产业规划和城乡基础设施规划，加大对县区特色产业、骨干企业和重点项目支持力度，推动县域经济加快发展。

　　用好开放发展平台。加强与“一带一路”、国际陆海贸易新通道、关中—天水经济区、关中平原城市群城市的经贸合作和人文交流，进一步强化合作的广度和深度，拓展开放的范围和层次。精心筹办好公祭伏羲大典、麦积山论坛、重大文化体育赛事等活动，积极争取开通西安至天水至张掖至敦煌航线。加大对外宣传推介力度，全面提升天水知名度和影响力。扩大对外贸易，设立天水海关，力争开通国际陆海贸易新通道货运班列天水农产品专列，争取在阿联酋迪拜设立天水花牛苹果展示中心，推广运用中国国际贸易“单一窗口”国家标准版，加快完成跨境电商公共服务平台数据联通和监管场地运营，实现我市跨境电商本地通关，全年完成进出口总值40亿元以上。

　　支持发展民营经济。坚持“两个毫不动摇”，认真贯彻中央、省上支持民营经济和中小微企业发展的决策部署，促进民营经济和中小微企业发展。加快推进市场准入负面清单制度，取消歧视性附加条件和隐性条款，营造公平开放的市场准入环境。加大政策扶持力度，全面落实省上促进中小微企业高质量发展58条和市上支持非公经济发展21条政策措施，降低土地、物流、人才、税费等要素成本。强化服务保障，建立完善领导干部联系制度，开展结队帮扶服务行动，为民营企业和中小微企业提供“保姆式”“贴近式”跟踪服务。加强金政企对接，完善融资担保体系，加大信贷投放力度，破解民营企业和中小微企业融资难、融资贵问题。持续推进非公经济“梯级培育、转型升级”行动，加强“四上”企业培育统计，抓好“个转企”“小升规”工作，年内培育“个转企”550户以上。

　　（八）努力提高保障改善民生水平

　　以满足人民日益增长的美好生活需要为目标，深入实施民生保障工程，加快完善公共服务体系，不断提高人民群众的生活水平和质量。

　　提高就业创业质量。坚持把稳就业摆在突出位置，认真落实各项就业创业扶持政策，扶持普通高校毕业生到企业、到基层就业，引导鼓励大学生、农民工等返乡人员就业创业，年内新增城镇就业5万人以上。优化创业服务方式，提升创业孵化基地、创业园区和就业见习基地建设质量和层次。及时化解各类劳动争议，解决好农民工工资拖欠问题，构建和谐劳动关系。重视做好创新型、高层次、急需紧缺人才的引进、管理和使用，营造吸引人才、留住人才、用好人才的氛围。

　　促进社会事业发展。坚持教育优先发展战略，统筹推进城乡义务教育一体化发展，实施全面改薄、农村小规模学校和寄宿制学校建设项目270个，进一步消除城区“大班额”，确保秦安、清水两县通过国家义务教育发展基本均衡县验收。继续实施第三期学前教育行动计划，全面推广农村小学小班教学模式创新行动计划，推进高中阶段教育普及攻坚，加快天水职教园区建设，推动天水师范学院升格天水师范大学。深入推进“健康天水”建设，深化医疗卫生体制改革，建立完善医疗卫生行业综合监管协调机制，加大基层全科医生培养力度，加快中医中药产业发展，提升医疗卫生服务能力和水平。大力发展文化体育事业，继续做好“三馆一站”免费开放，加强文化遗产保护利用。广泛开展全民健身活动，实施乡镇及社区体育中心8处、村级农民健身工程90处，完成南山体育场改造提升工程，市体育中心上半年投入使用。

　　加大社会保障力度。完善多主体供给、多渠道保障、租购并举的住房制度，满足群众多层次住房需求，实施北园子、东团庄、火柴厂、罗玉新村、翠山新村等片区棚户区改造项目42个。推进社会救助与脱贫攻坚有效衔接，提高城乡低保补助水平和特困人员救助供养补助标准，大力发展社会福利和慈善事业，重视城镇困难职工解困脱困，做好农村留守妇女、儿童和空巢老人、特困群体关爱服务工作。加快麦积全国综合养老示范基地建设，规范城市日间照料中心等养老机构管理，提升养老服务水平。重视殡葬改革和服务设施建设，加快建设翠山公墓区、盘龙山殡仪馆。持续抓好城镇职工养老、工伤、失业等社保经办管理，高度重视困难企业和困难职工的社保问题，落实好失业保险援企稳岗、被征地农民养老保险政策，做好社会保险待遇提标工作，加强社会保险稽核管理，确保社保基金安全可持续。

　　创新加强社会治理。重视地方立法工作，认真做好《天水市城区引洮供水设施保护条例（草案）》的起草和提请审议，加强政府规章的制定和实施。统筹优化法治宣传、律师公证、人民调解、法律援助等法律服务资源，健全公共法律服务体系。加强应急管理能力建设，提升全市防灾减灾、应急救援和突发事故处置能力。扎实开展安全生产大检查、安全风险分级管控和隐患排查治理，坚决杜绝重大安全生产事故发生。深入推进食品药品监管能力建设和突出问题治理，开展食品安全示范城市全域创建工作，确保广大群众饮食用药安全。积极借鉴“枫桥经验”，健全城乡社区治理体系，完善预防和化解社会矛盾机制，落实信访工作责任制，办好“市委书记市长留言板”“市长信箱”。做好退役军人工作，深化双拥共建，争创全国双拥模范城“六连冠”。深化民族团结进步创建工作，依法加强宗教事务管理，促进民族团结、宗教和顺。纵深推进扫黑除恶专项斗争，深入开展打击治理电信网络新型违法犯罪专项行动，继续实施“雪亮工程”，健全完善立体化信息化社会治安防控体系。支持工会、共青团、妇联等人民团体发挥党和政府联系广大群众的桥梁纽带作用。认真做好统计调查、新闻出版、外事侨务、人民防空、气象地震、档案史志、老年教育、残疾人事业、关心下一代等工作。

　　坚持办好民生实事。在全面完成省政府民生实事的同时，紧盯广大群众最关心、最直接、最现实的问题，集中力量办好10件实事。（1）全面完成农村饮水安全巩固提升工程，解决21.38万户98.93万人饮水不稳定问题。（2）实施棚户区改造1.6万户，改造农村C级危房3360户。（3）实施城中村、城郊村和社区改灶、改炕、改炉、优质煤炭补贴“三改一补”4万户。（4）深入实施“厕所革命”，新改建城区厕所20座、旅游景区厕所65座，实施农村厕所改造提升18685户。（5）引导高校毕业生到企业就业1000人，加强基层企业人才储备。（6）建成人民公园、天水电大后院立体停车库和天河广场、岷山生态文化公园地下停车场等，新增城区停车位3000个。（7）新建城区人行过街天桥2座、藉河跨河大桥连接藉河风情线便桥3处，整修城区主次干道10万平方米。（8）新建市区社区便民市场5处。（9）新建、改造商业餐饮街区10处，大力发展夜间经济，提升特色餐饮发展水平。（10）全面疏浚治理河道7条，改善水域生态环境，预防减少暴洪灾害。

　　三、加强政府自身建设

　　新形势新任务对政府自身建设提出了新的更高要求。我们将对标忠诚干净担当、为民务实清廉，全面加强政府自身建设，努力建设人民满意的政府。

　　进一步强化政治意识。坚持把政治建设摆在首位，树牢“四个意识”，坚定“四个自信”，坚决做到“两个维护”，始终在政治立场、政治方向、政治原则、政治道路上同以习近平同志为核心的党中央保持高度一致。严守政治纪律和政治规矩，自觉把讲政治贯穿于政府工作的各个方面，体现在做决策、想问题、抓落实的全过程，坚决贯彻党中央国务院、省委省政府和市委各项决策部署，确保政令畅通、令行禁止。

　　进一步强化学习教育。认真落实市政府党组理论中心组学习制度，推进“两学一做”学习教育常态化制度化，增强“八种本领”，做到“五个过硬”，加快学习型政府建设。加强政治理论学习，坚持读原著、学原文、悟原理，做到学而信、学而思、学而行，推动理论武装工作深入开展。加强方针政策学习，善于从国家政策中寻找机遇、挖掘机遇、把握机遇，研究政策的“含金量”，找准落实的“契合点”，及时把政策转化为具体发展举措。加强业务知识学习，力求学好“公共课”和“专业课”，用专业思维和专业方法谋划工作、解决问题、推动发展。

　　进一步强化为民情怀。积极践行以人民为中心的发展思想，心系群众冷暖，情牵百姓所需，千方百计做好富民利民惠民安民的工作。坚持“民有所呼、我有所应”，时刻牢记群众最盼最急最忧最怨的问题，始终紧盯群众心中的痛点、难点和堵点，从能够办得到的事情做起，从群众最期盼的问题入手，一件一件地解决，一项一项地落实，努力做到为人民服务、对人民负责、受人民监督、让人民满意。

　　进一步强化依法行政。加快法治政府建设，自觉接受市人大及其常委会、市政协和社会各界的监督，认真落实政府重大决策出台前向人大报告制度，提高人大代表建议和政协委员提案办理质量。严格执行市政府工作规则，健全决策咨询制度，落实法律顾问制度，坚持公众参与、专家论证、风险评估、合法性审查和集体讨论决定，推进政府决策民主化、科学化和法治化。加大政务公开和信息公开力度，完善新闻发布制度，保障群众知情权、参与权和监督权。

　　进一步强化担当尽责。坚持问题导向和目标导向，始终把扭住不放抓落实作为政府工作的生命线，严格执行省政府“十不准”规定，坚持以实为本、以真为要、以干为先，定下的事情马上就办，承诺的事项坚决兑现，部署的工作一抓到底，说到做到、不放空炮，着力提高政府执行力和公信力。深入开展不落实、落不实专项治理，紧紧盯住不落实的事情，跟踪督查不落实的单位，约谈问责不落实的干部，坚决治庸、治懒、治散、治慢，推动各项工作落细落小落实。健全完善清单管理制度和责任分解机制，对政府重点工作和重大项目，年初建账、年中查账、年底交账，确保每项工作有人抓、有人管、能落实。

　　进一步强化廉政建设。认真落实党组工作条例，把党的领导贯穿政府工作全过程，不断加强政府系统党的建设。严格履行管党治党主体责任和领导干部“一岗双责”，细化靠实责任，层层传导压力，深入推进政府系统党风廉政建设。严格落实中央“八项规定”及实施细则精神和省市《实施办法》，大力整治“四风”新表现新动向，坚决反对形式主义、官僚主义，着力解决表态多调门高、行动少落实差等突出问题。健全权力运行制约和监督机制，以公共资金、公共资产、公共资源管理为重点，依法加强审计监督，严肃查处违纪违法案件，推动各类巡视巡察督查反馈问题整改，坚决防止和纠正损害群众利益的不正之风，让清廉从政、清正干事成为政府工作的鲜明底色。

　　各位代表，新的目标展现美好前景，新的使命更加催人奋进。我们要紧密团结在以习近平同志为核心的党中央周围，在省委、省政府和市委的坚强领导下，不忘初心、牢记使命，大力弘扬敢打必胜、舍我其谁的拼搏精神，始终保持永不懈怠、一往无前的奋斗姿态，为加快全面建成小康社会进程、建设幸福美好新天水努力工作，以优异成绩向中华人民共和国成立70周年献礼！
